# Supplementary material for: Significance of gene variants for the functional biogeography of the near-surface Atlantic Ocean microbiome
Source: Nat Commun. 2022 Jan 24;13:456. doi: 10.1038/s41467-022-28128-8 (PMC8786918; doi:10.1038/s41467-022-28128-8)
Supplement: Supplementary file 1 — Supplementary Information [file 41467_2022_28128_MOESM1_ESM.pdf]

Significance of gene variants for the functional biogeography of the near-surface Atlantic Ocean microbiome

Leon Dlugosch[1], Anja Poehlein[2], Bernd Wemheuer[2], Birgit Pfeiffer[2], Thomas H.Badewien[1], Rolf Daniel[2], Meinhard Simon[1,3]

[1] Institute for Chemistry and Biology of the Marine Environment, University of Oldenburg,

Carl von Ossietzky Str. 9-11, D-26129 Oldenburg, Germany

[2] Department of Genomic and Applied Microbiology and Göttingen Genomics Laboratory, Institute of

Microbiology and Genetics, Georg-August University of Göttingen,

Grisebachstr. 8, D-37077 Göttingen, Germany

[3] Helmholtz Institute for Functional Marine Biodiversity at the University of Oldenburg (HIFMB) Ammerländer Heerstraße 231, D-26129 Oldenburg, Germany

Supplementary Information

Table 1 Location and hydrographic properties of the stations along the transect.

Table 2 Sequencing statistics.

Table 3 Richness, diversity and evenness of taxa, KOs and non-redundant genes.

Supplementary Data 1 Log-fold change (l2) and Benjamini-Hochberg adjusted p-values of differential abundance (DESeq2) analysis of selected pathways (amino acid, oligo- and monosaccharide transporters, nitrogen metabolism, vitamin B12 and B1 synthesis, phytosynthesis and CAZymes) between functional profile clusters 1, 2 and 3 of the AOM. (separate Excel file)

Supplementary Data 2

1. Taxonomy of 158 species/genomes and their temperature range, temperature of maximum abundance and lowest and highest temperature of occurrence
2. Number of variants, temperature range and standard deviation of highly abundant KOs of prominent taxa of the AOM. (separate Excel file).

Fig. 1 Collectors curves of richness of gene sequences, KOs and taxa of the AOM.

Fig. 2 A, Contour plots of potential temperature, salinity and density of the upper 200 m along the Atlantic Ocean transect; B, temperature-salinity plot of the water masses of the upper 200 m along the transect.

Fig. 3 Hydrographic, biotic and biogeochemical properties of the Southern and Atlantic Ocean during the RV Polarstern cruises ANTXXVIII/4 and -/5.

Fig. 4 Taxonomic composition of the AOM.

Fig. 5 Determination and validation of clusters.

Fig. 6 Differential abundance of KOs and CAZymes.

Fig. 7 CAZyme abundance cluster dendrogram

Fig. 8 Taxonomic breakdown of CAZyme abundances.

Fig. 9 Normalized abundance of clusters of gene variants of prominent taxa of the AOM in the biogeographic provinces along the transect at stations 193 to 330.

Fig. 10 Temperature ranges of prominent species of the AOM.

Supplementary Table 1

Environmental and biotic data of stations of cruises ANTXXVIII/4 and -/5 at 20 m depth. Chl *a*: chlorophyll *a*; BPP: bacterial biomass production; POC: particulate organic carbon; TPN: total particulate nitrogen. Annual mean concentrations of phosphate and nitrate were extracted from the World Ocean Atlas 2018, provided by the National Oceanic and Atmospheric Administration (https://www.ncei.noaa.gov/access/world-ocean-atlas-2018/). na: not available; *: linearly interpolated

Station	Latitude [°N]	Longitude [°W]	Date	Province	Temperature [°C]	Salinity	Chl *a* [µg L^{-1}]	Prokaryotes [10^{5} cells ml^{-1}]	BPP [ngC L^{-1} h^{-1}]	POC [µg L^{-1}]	TPN [µg L^{-1}]	Nitrate [µM]	Phosphate [µM]
**193**	-61.717	*55.167*	2012-03-19	APLR	1.225	34.195	0.80	8.42	145.0	116	25	25.728	1.819
**241**	-61.244	*57.074*	2012-03-25	APLR	1.226	34.043	1.19	10.60	21.2	114	26	24.709	1.788
**287**	-60.500	*55.500*	2012-04-04	ANTA	1.832	33.837	1.23	2.16	18.1	148	3	24.881	1.687
**179**	-57.983	*59.867*	2012-03-16	ANTA	4.392	33.707	0.08	4.19	13.5	38	7	22.146	1.574
**178**	-56.170	*62.633*	2012-03-16	SANT	6.981	33.979	0.35	8.51	*na*	61	12	16.317*	1.548
**296**	-51.054	*66.459*	2012-04-11	FKLD	8.148	33.001	0.40	12.90	70.0	83	14	10.488	1.280
**297**	-47.941	*61.923*	2012-04-12	FKLD	10.344	33.492	0.57	19.00	233.2	105	22	12.017	1.049
**300**	-39.798	*50.577*	2012-04-15	SATL	19.484	35.311	0.30	8.04	186.4	60	11	3.901	0.455
**302**	-34.239	*42.965*	2012-04-17	SATL	22.131	35.843	0.15	5.36	152.8	42	6	0.040	0.010
**308**	-21.229	*35.402*	2012-04-21	SATL	27.149	37.316	0.10	6.39	98.7	33	6	0.554	0.059
**310**	-18.664	*33.725*	2012-04-22	SATL	27.371	37.394	0.11	7.53	256.5	37	6	0.126	0.094
**311**	-15.522	*31.862*	2012-04-23	SATL	26.882	37.240	0.08*	5.93	*na*	33.5*	*na*	0.247	0.029
**312**	-11.898	*29.752*	2012-04-24	SATL	27.270	37.014	0.06	6.26	163.0	30	6	0.300	0.096*
**313**	-8.211	*27.991*	2012-04-25	SATL	28.012	36.285	0.09	7.88	276.1	34	6	0.050	0.162
**315**	-1.487	*25.318*	2012-04-27	WRTA	27.826	35.977	0.33	8.66	178.5	57	11	0.038	0.064
**319**	12.590	*22.196*	2012-05-01	NATR	22.923	35.776	0.72	12.90	191.9	60.5*	*na*	0.155*	0.157
**320**	16.904	*21.569*	2012-05-02	NATR	21.415	36.206	0.51	6.94	351.4	64	12	0.272	0.141
**321**	20.705	*21.170*	2012-05-03	NATR	20.770	36.815	0.45	9.78	102.1	71	14	0.855	0.098
**324**	33.398	*13.534*	2012-05-07	NAST	17.787	36.606	0.15	7.22	134.8	54	9	0.885*	0.076*
**326**	35.239	*12.897*	2012-05-08	NAST	17.918	36.619	0.21	6.27	110.2	65	9	0.915*	0.053
**329**	43.042	*10.935*	2012-05-10	NADR	13.744	35.786	0.74	10.50	108.8	101	19	0.945	0.067
**330**	47.0438	8.0905	2012-05-11	NADR	12.579	35.6486	1.90	14.90	373.0	254	52	3.179	0.234

Supplementary Table 2

Statistics of sequencing, assembly and ORF-prediction of the AOM samples of all stations visited and analyzed. *: based on contigs ≥ 210 bp contigs and ORFs, **: based on contigs ≥ 500 bp, *** genes not included in OM-RGC_v2 (for details see materials and methods of the publication).

Station	*Total bp*	*Joined reads*	*Contigs**	*Contigs***	*N50***	*Total assembly length [bp]**	*Largest contig [bp]*	*Predicted ORFs*	*Novel genes****	*Mapped reads*
193	7,435,000,546	12,427,245	1,501,196	183,054	1,654	606,362,663	115,786	417,100	46,554	9,510,812
241	6,071,598,618	10,027,320	962,824	116,387	1,571	387,239,824	325,929	268,873	47,990	7,301,266
287	6,322,636,050	9,471,523	1,434,266	183,574	1,126	555,042,209	258,892	333,526	121,003	6,166,447
179	6,198,682,756	7,599,385	971,371	143,473	1,874	436,536,877	262,003	290,543	29,190	6,139,726
178	6,064,805,297	8,176,910	1,300,260	130,039	1,509	486,212,585	306,237	287,516	33,687	5,937,808
296	19,592,095,808	26,002,512	2,505,074	427,057	1,894	1,203,911,331	256,578	100,1702	173,277	21,787,506
297	6,616,159,289	9,041,391	1,357,387	198,801	1,617	585,795,325	162,636	441,554	54,874	7,343,246
300	6,792,243,498	12,739,153	1,582,103	360,444	877	780,657,750	143,583	567,271	48,919	9,788,569
302	5,503,346,589	11,099,908	1,398,405	322,151	807	675,992,337	81,215	258,162	18,012	7,599,287
308	4,841,364,911	8,620,757	1,087,405	263,756	1,044	578,609,656	152,476	388,272	24,475	6,361,388
310	6,517,496,619	11,188,810	1,220,934	292,443	1,033	637,971,540	93,510	494,727	29,117	8,857,004
311	7,559,399,537	11,548,284	1,276,149	323,727	1,029	676,526,273	154,832	549,470	33,950	9,604,172
312	4,447,667,609	7,750,178	1,055,905	241,878	1,040	550,785,256	125,412	368,056	36,673	5,431,202
313	7,892,259,228	13,906,649	1,416,050	310,566	1,068	729,545,280	782,123	548,478	40,407	10,839,614
315	6,949,599,597	12,187,168	1,305,054	309,827	1,028	684,170,427	659,572	520,142	30,874	9,499,620
319	6,984,066,868	12,396,808	1,352,845	342,885	1,114	742,215,671	144,779	593,845	69,417	10,138,608
320	7,501,417,624	12,965,323	1,325,747	337,799	1,102	725,591,569	889,385	556,011	63,999	10,668,816
321	8,541,115,749	14,587,332	1,751,299	451,148	1,027	942,741,816	659,617	699,588	76,855	11,603,701
324	5,304,775,998	13,853,045	717,695	140,680	890	341,757,754	538,303	325,162	19,512	9,635,236
326	21,965,269,770	33,849,158	3,428,669	841,029	1,150	1,867,599,514	266,735	1,291,360	469,340	26,396,598
329	16,064,549,937	22,926,879	2,629,046	598,340	1,064	1,381,208,536	157,398	859,230	167,630	18,165,412
330	21,005,691,360	28,029,489	3,151,195	724,155	1,115	1,675,633,057	264,112	996,361	350,612	21,744,446

Supplementary Table 3

Richness and inv. Shannon index of taxa, KEGG orthologues and non-redundant gene sequences of the samples of all stations visited. Prior to analysis samples were rarefied to 2 Million reads per sample.

Station	Taxon Richness	Taxon Inv. Shannon index	Taxon Evenness	KO Richness	KO Inv. Shannon index	KO Evenness	nr-sequence Richness	nr-sequence Inv. Shannon index	nr-sequence Evenness
193	11,279	281.6560	0.604530	7,636	2077.083	0.854383	388,767	152097.8	0.927086
241	9,234	426.9284	0.663328	6,227	1566.834	0.842063	233,580	68220.0	0.900432
287	10,346	449.3945	0.660717	6,986	1661.252	0.837733	239,732	67701.5	0.897926
179	10,091	383.3060	0.645252	6,510	1590.384	0.839500	243,101	76958.2	0.907246
178	10,185	260.4246	0.602721	6,863	1535.687	0.830520	275,340	85570.1	0.906699
296	12,480	548.7825	0.668764	8,045	1972.917	0.843704	430,193	165089.4	0.926168
297	11,931	543.4357	0.670926	7,927	1877.380	0.839564	437,563	185316.7	0.933855
300	12,705	328.3156	0.613135	8,079	1881.473	0.838034	725,790	379280.4	0.951909
302	12,196	285.3285	0.600885	6,989	1509.619	0.826879	554,253	193322.5	0.920361
308	11,841	347.0930	0.623669	6,388	1442.888	0.830205	468,850	160741.2	0.918021
310	11,648	240.7508	0.585691	6,263	1412.486	0.829646	467,964	146765.4	0.911187
311	11,301	231.7185	0.583492	5,736	1358.304	0.833552	451,703	152627.7	0.916670
312	11,018	278.3843	0.604795	6,418	1446.299	0.830031	369,173	126256.2	0.916300
313	11,651	235.5073	0.583323	6,182	1473.013	0.835689	460,697	140057.5	0.908693
315	11,976	355.6772	0.625518	6,821	1631.444	0.837950	507,783	198160.1	0.928376
319	11,801	453.9574	0.652522	7,778	1652.684	0.827114	548,276	260992.7	0.943828
320	11,760	503.3809	0.663790	8,033	1975.159	0.843971	501,941	191934.0	0.926763
321	12,515	446.4145	0.646683	8,134	1810.951	0.833159	628,164	298974.2	0.944389
324	11,560	208.3869	0.570735	7,895	1655.606	0.825934	577,276	215905.7	0.925865
326	12,090	324.2307	0.615040	8,762	2167.087	0.846110	557,520	240720.6	0.936524
329	11,705	364.6921	0.629718	8,318	2096.678	0.847325	528,128	222835.0	0.934515
330	11,321	275.1644	0.601791	8,091	2460.928	0.867732	422,390	150352.0	0.920258

For Supplementary Data 1 and 2 see extra Excel files.

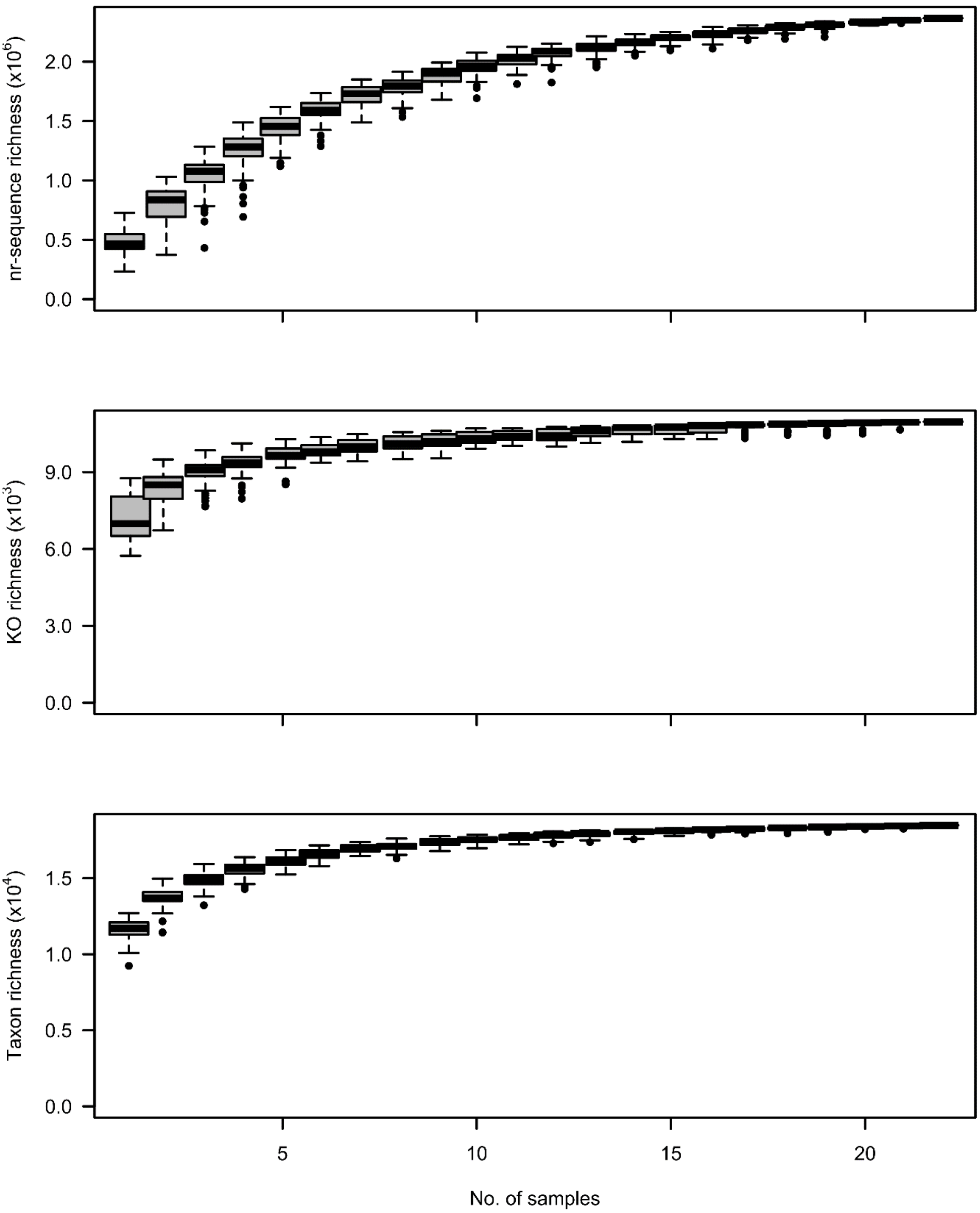

Supplementary Figure 1: Collectors curves of richness of non-redundant (nr) gene sequences, KEGG orthologues (KO) and taxa. For the calculation of collectors curves only taxonomically and functionally classified genes were from 22 samples were used. Samples of nr-sequence, KO and taxonomic profile where rarefied to 2 Million reads prior to collectors curve calculation (100 permutations). Median values represented by vertical lines, interquartile ranges shown as boxes, whiskers extending up to 1.5 times the interquartile range and points show outliers

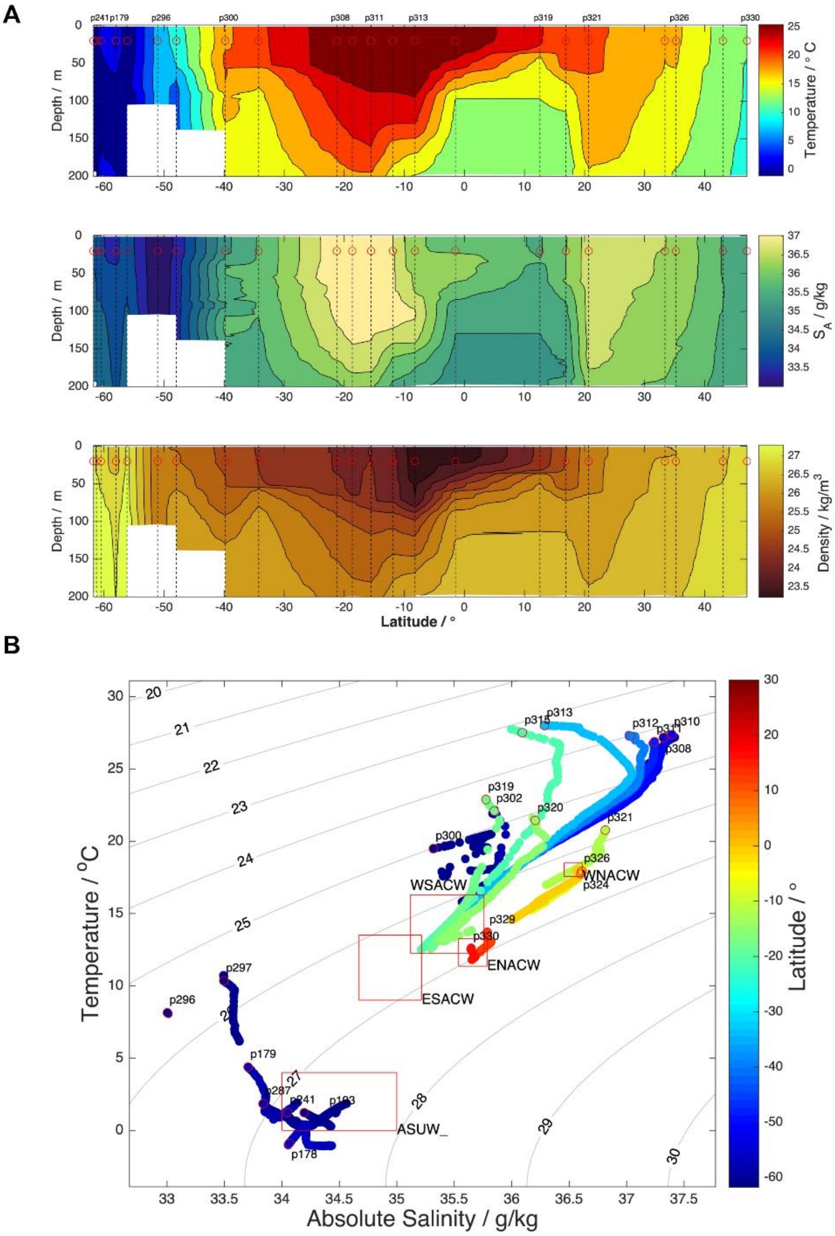

Supplementary Figure 2: Contour plots of hydrographic features and temperature versus salinity of the water masses of the upper 200 m along the transect of RV Polarstern cuises ANTXXVIII/4 and -/5 **A**: Contour plots of potential temperature, salinity and density of the upper 200 m along the transect of the Southern and Atlantic Ocean between 62°S and 47°N investigated for the AOM. Plots are based on continuous measurements of the CTD probes at the visited stations (vertical dotted lines). Numbers above panel A indicate stations. Red circles mark the depth of sampling (20 m). **B**: Temperature-salinity plot of the water masses of the upper 200 m along this transect. Color code indicates latitude from 62°S to 47°N and numbers the stations visited (for details see Table S1). Red squares identify major water masses in the Atlantic and Southern Ocean. AASW: Antarctic Surface Water; ACW: Antarctic Circumpolar Water; WW: Winter Water; ESACW: East South Atlantic Central Water; ENACW: East North Atlantic Central Water; WNACW: Western North Atlantic Central Water; WSACW; Western South Atlantic Central Water.

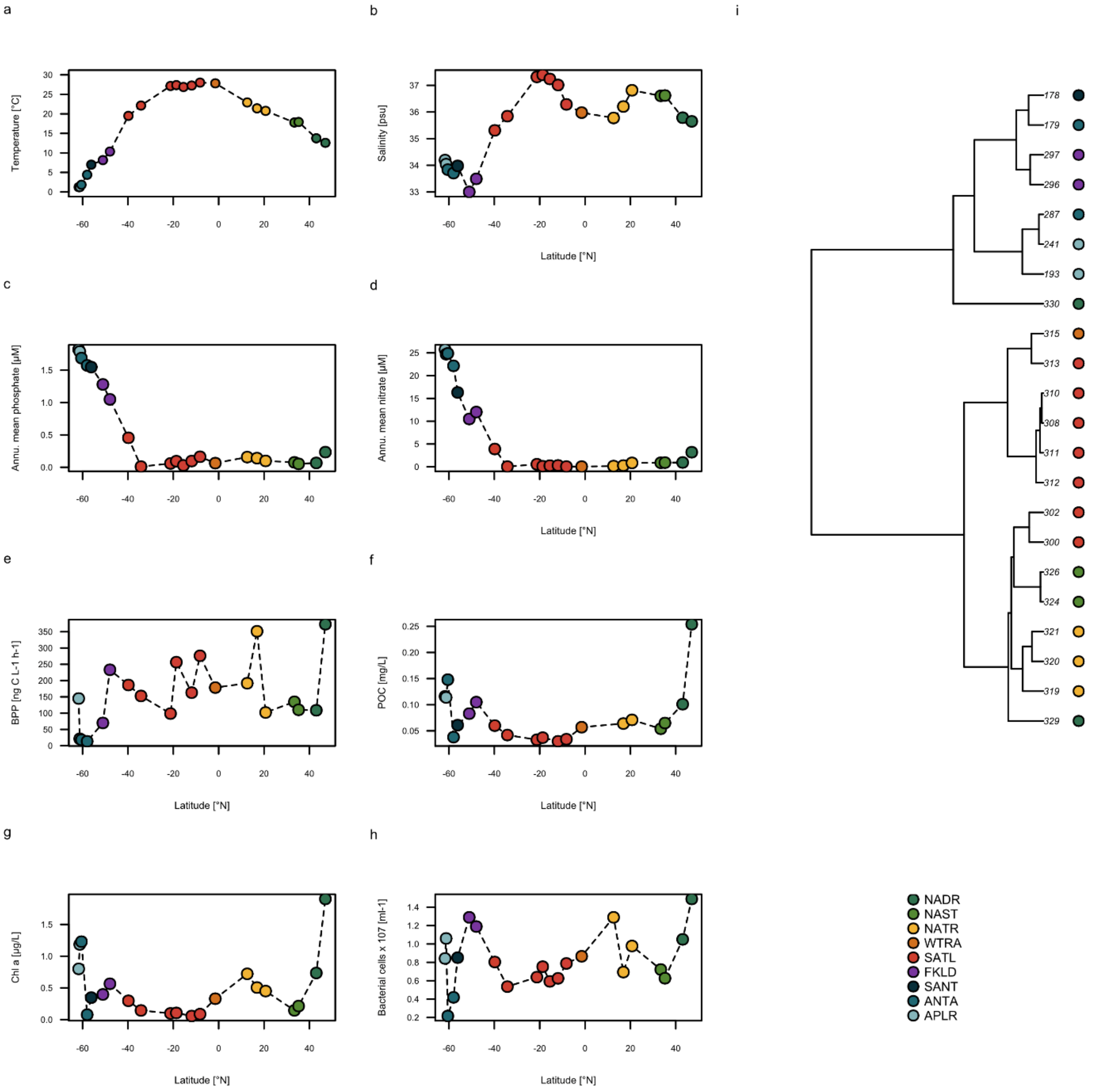

Supplementary Figure 3: Hydrographic, biotic and biogeochemical properties in the Southern and Atlantic Ocean during cruises ANTXXVIII/4 and -/5 with RV Polarstern. a-h: Temperature, salinity, annual mean phosphate, annual mean nitrate, bacterial biomass production, Particulate organic carbon (POC) chlorophyll *a* (Chl *a*) and bacterial cell counts at stations 193 to 330 along the Southern and Atlantic Ocean transect from 62°S to 47°N. **i**: Cluster analysis of stations 193 to 330 along the Southern and Atlantic Ocean transect from 62°S to 47°N considering in situ temperature, salinity and Chl *a* to identify oceanic provinces according to their hydrographic properties and position. For location details of stations see Fig. 1 and Table S1.

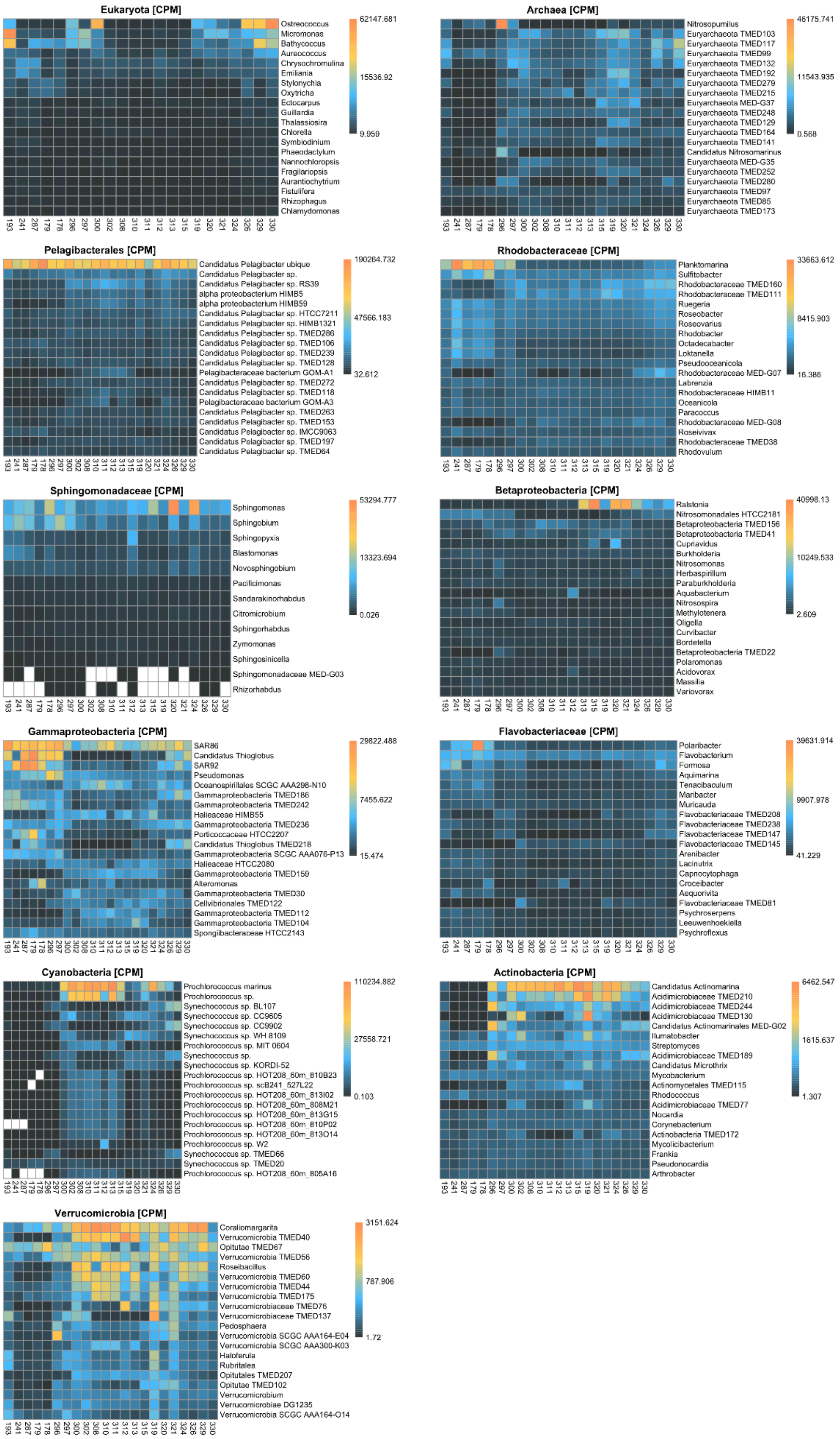

Supplementary Figure 4: Taxonomic composition of the AOM. Major phylogenetic groups and sublineages of these groups at stations 193 to 300 between 62°S and 47°N. For location of stations see Fig. 1 and Table S1.

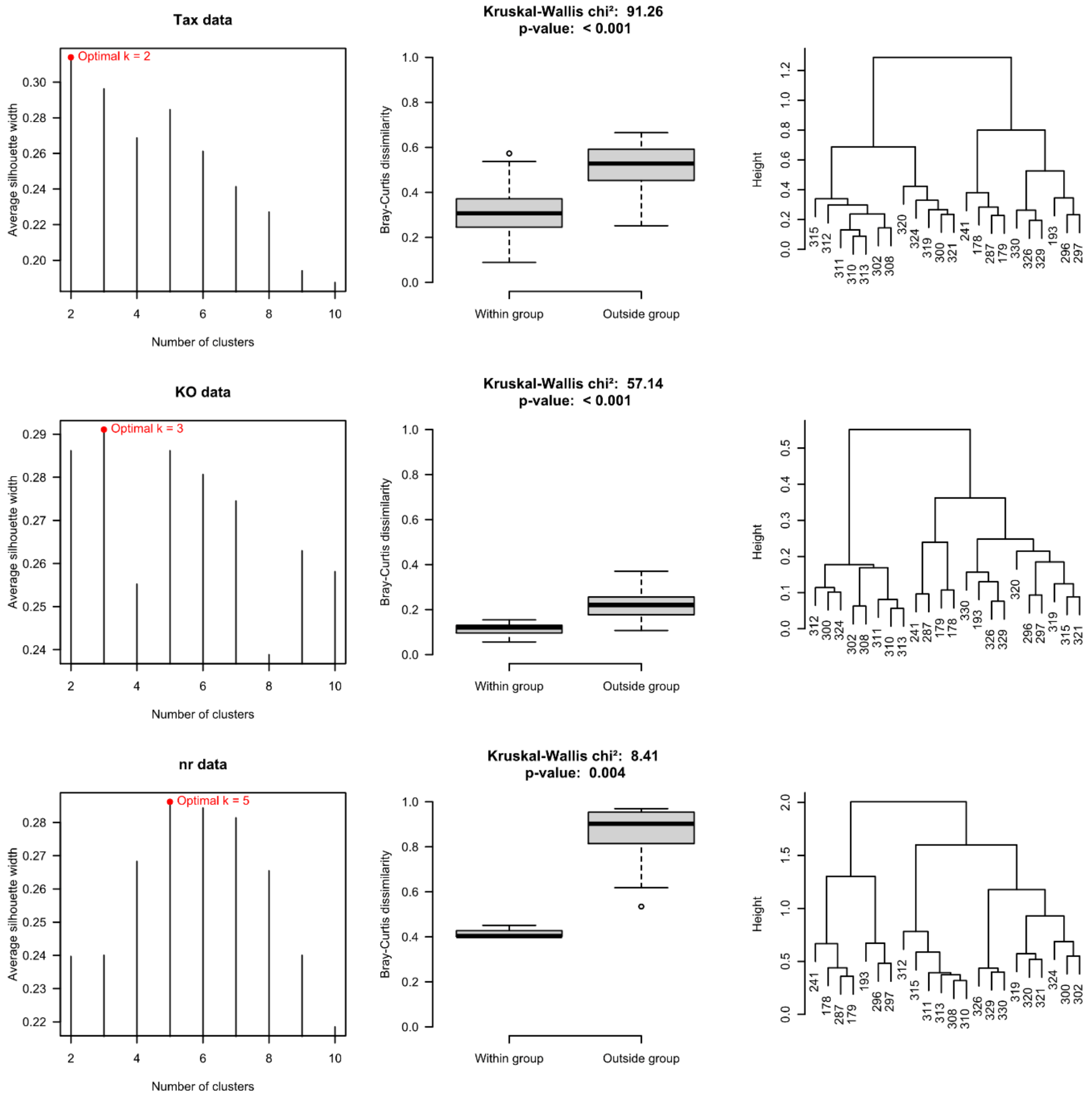

Supplementary Figure 5: Determination and validation of clusters. Optimal number of clusters determined by clusters silhouette coefficient. For validation, within cluster Bray-Curtis dissimilarity and distance to samples not included in clusters were tested using the non-parametric Kruskal-Wallis test. Median values represented by vertical lines, interquartile ranges shown as boxes, whiskers extending up to 1.5 times the interquartile range and points show outliers

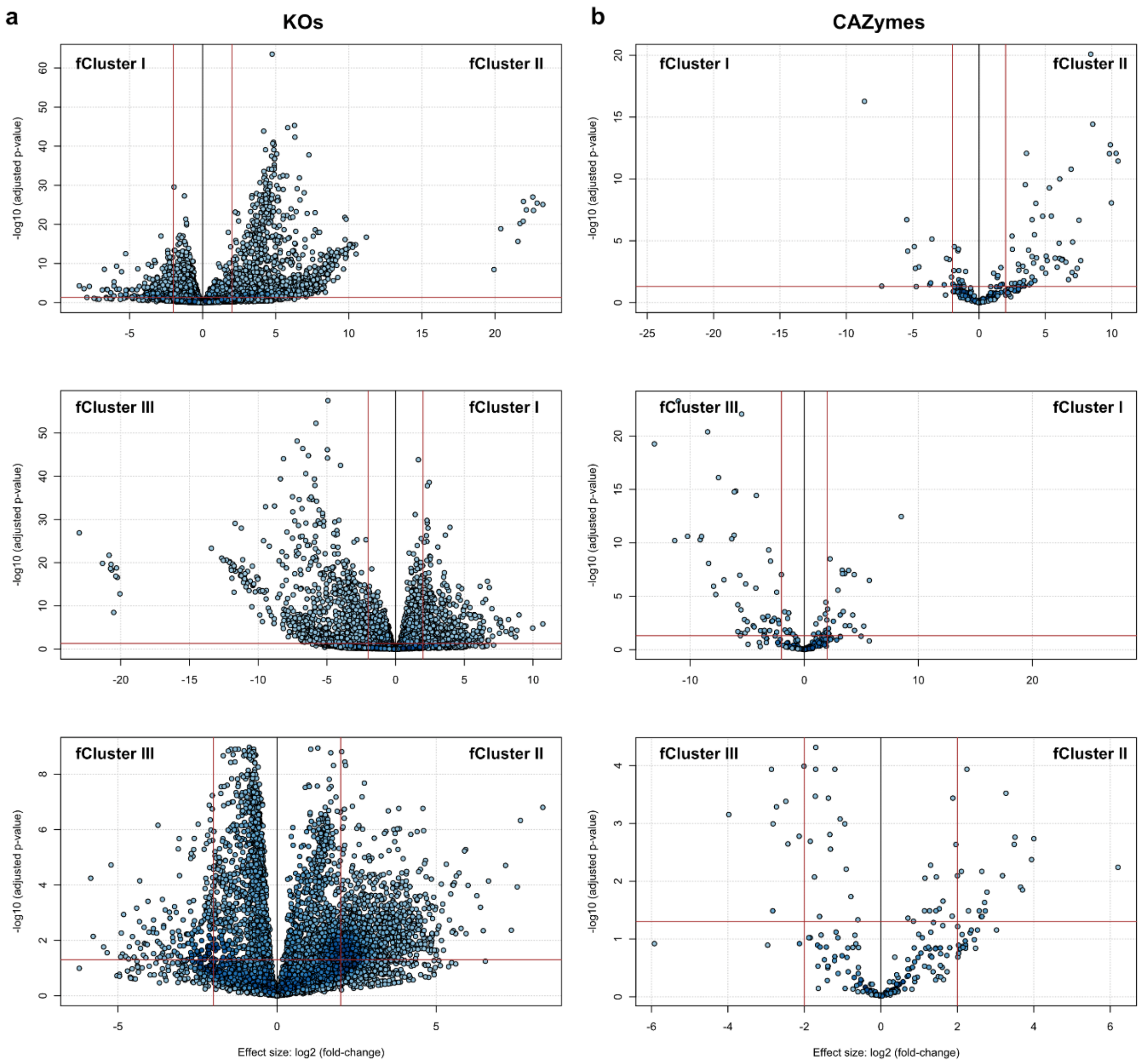

Supplementary Figure 6: Differential abundance of KOs and CAZymes Differential abundance of KOs and CAZymes between functional clusters of the AOM determined using DESeq2. Red lines indicate a Benjamini-Hochberg adjusted p-value ≤ 0.05 and a log2-fold change ≥ 2

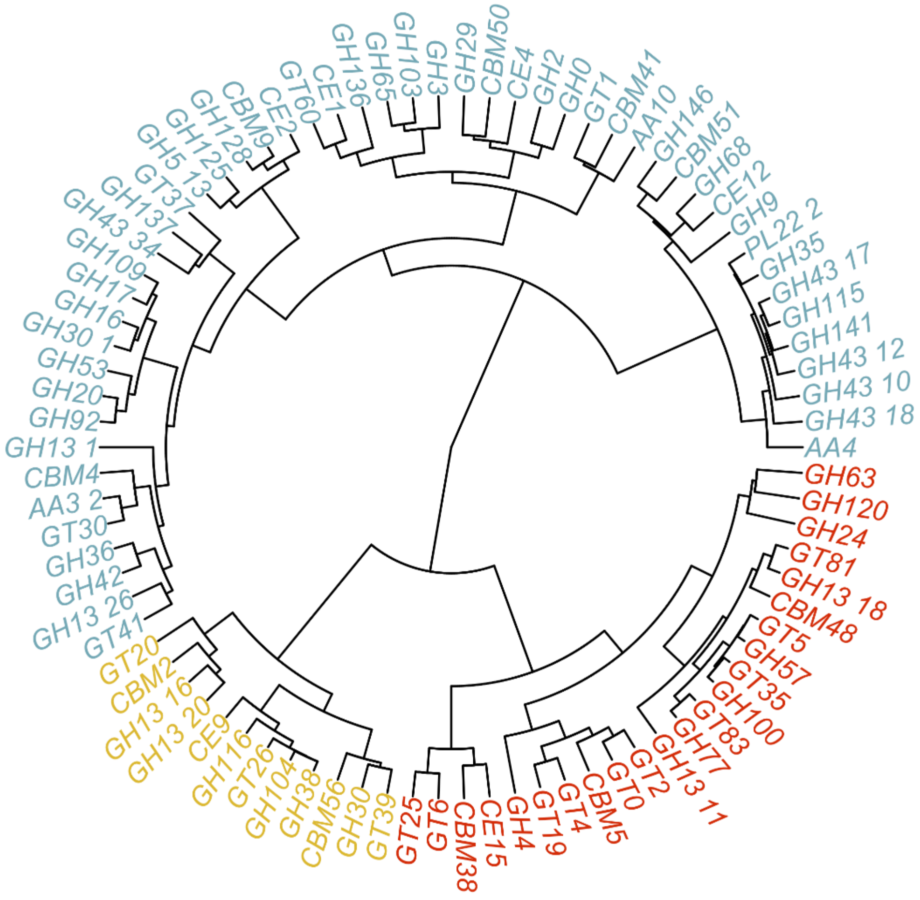

Supplementary Figure 7: CAZyme abundance cluster dendrogram Cluster analysis of normalized temperature abundance profiles of temperature-correlated CAZyme families using Euclidean distance between CAZyme families and Ward.D2 clustering to construct the dendrogram.

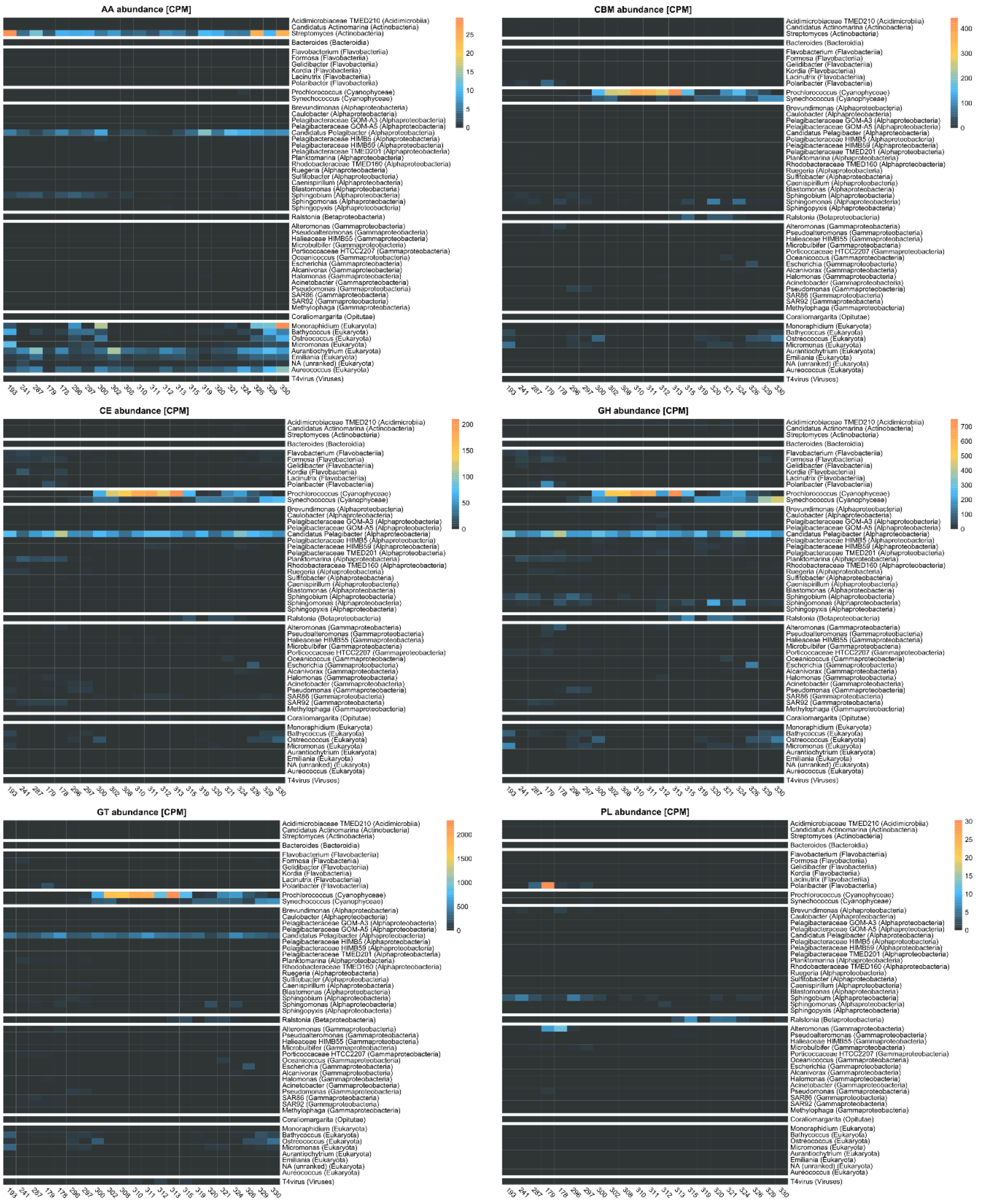

Supplementary Figure 8: Taxonomic breakdown of CAZyme abundances (AA: Auxiliary Activities, CBM: Carbohydrate Binding Modules, CE: Carbohydrate Esterases, GH: Glycoside Hydrolases, GT: Glycoside Transferases, PL: Polysaccharide Lyases) at stations 193 to 330 along the Southern and Atlantic Ocean transect between 62°S and 47°N. Data are given in counts per million (CPM) per station. For stations details see Fig. 1 and Supplementary Table 1.

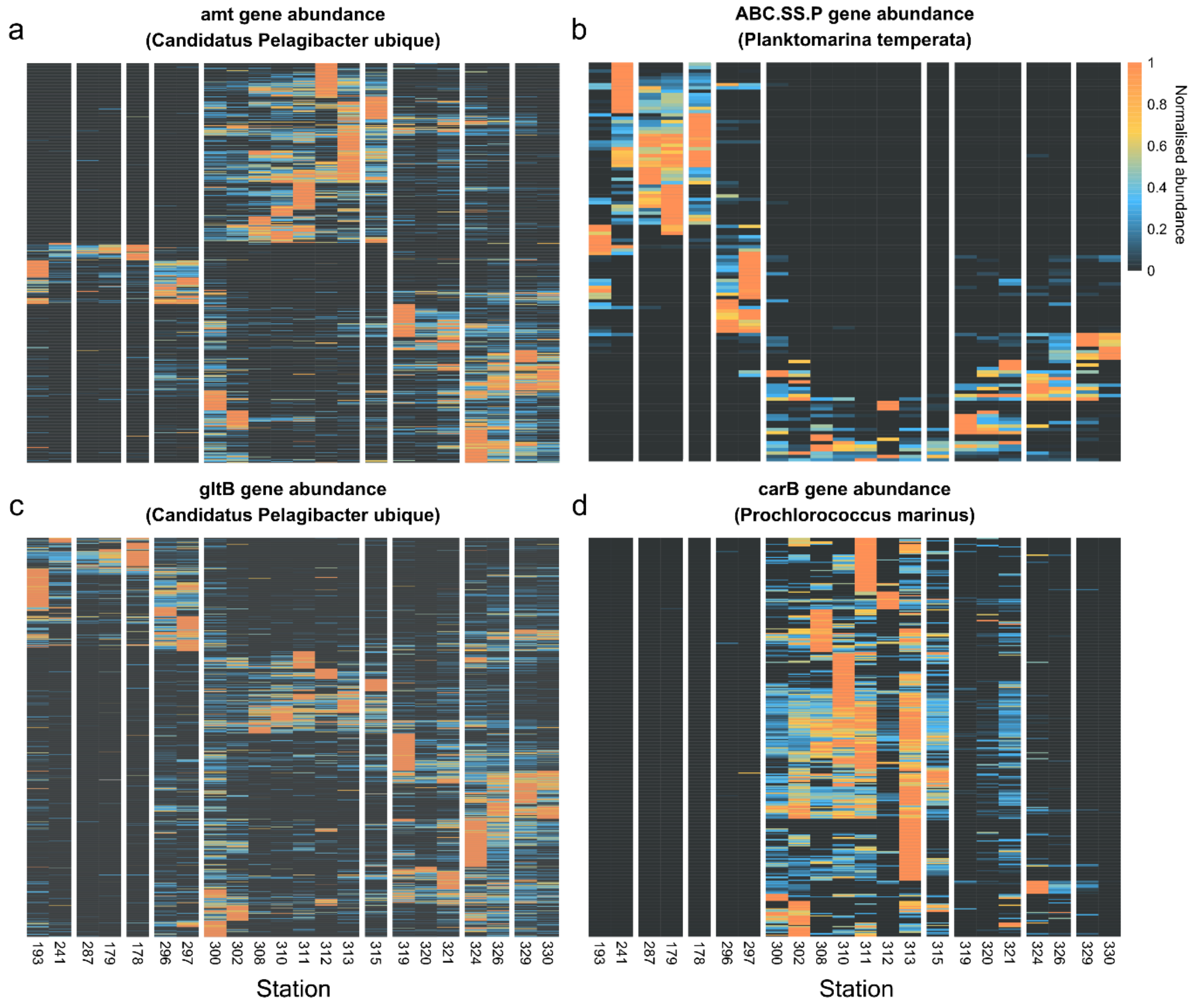

Supplementary Figure 9: Normalized abundance of clusters of gene variants of prominent taxa of the AOM in the biogeographic provinces along the transect at stations 193 to 330. **a**, Ammonium transporter of *Cand*. Pelagibacter ubique (KO3320 (*amt*); **b**, ABC sugar transporter (K02057/ABC.SS.P) of *Planktomarina temperate;* **c**, Glutamate synthase of *Cand*. Pelagibacter ubique (large subunit K00265 (*gltB*); **d**: carbamoyl-phosphate-synthase (large subunit, K01955/*carB*) of the pyrimidine metabolism of *Prochlorococcus marinus*; Gaps delineate biogeographic provinces. For station details see Fig. 1 and Table S1.

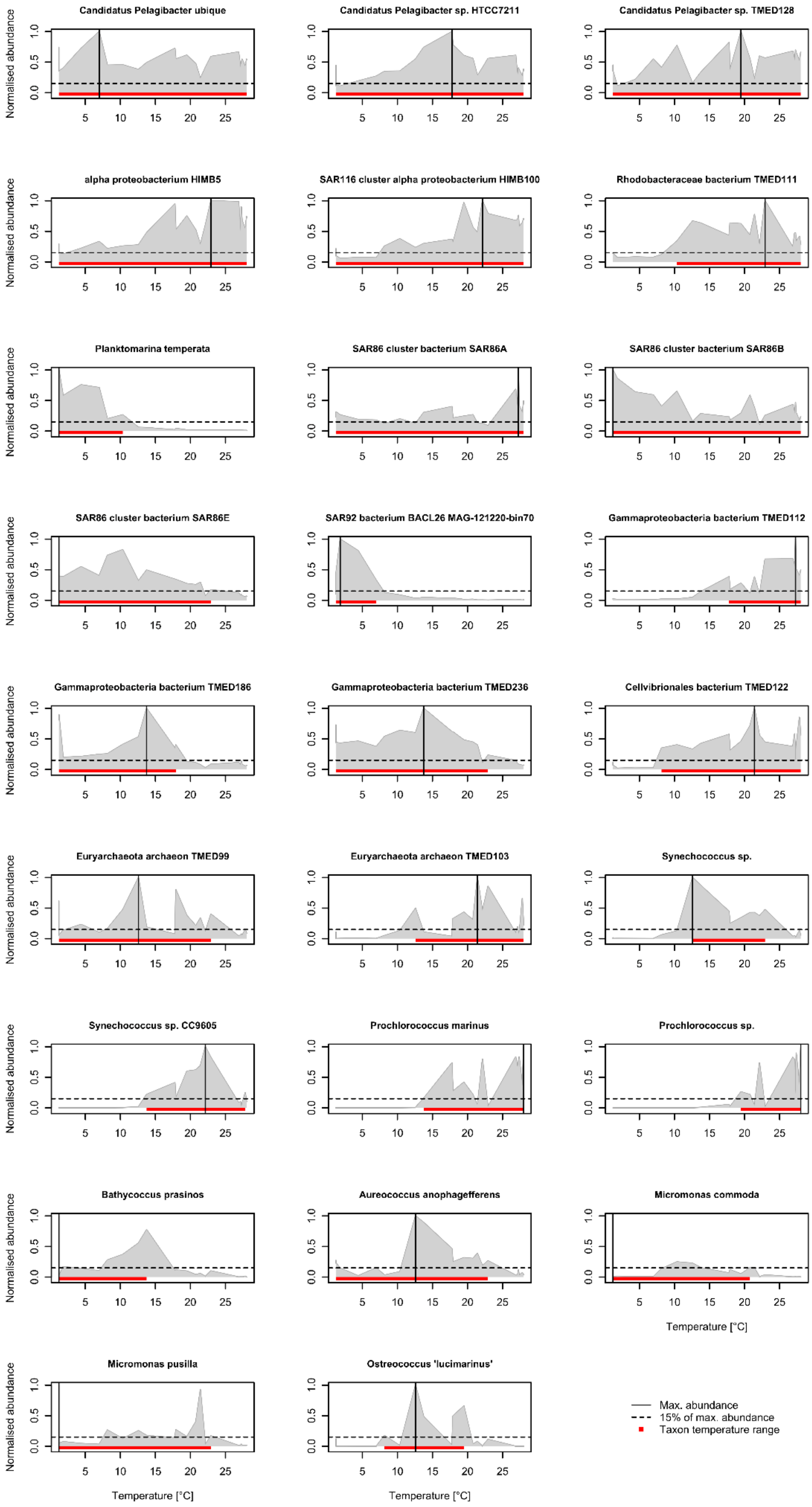

Supplementary Figure 10: Temperature ranges of prominent species of the AOM. Temperature range is defined as the temperature at which species occur with at least 15% of highest sequence abundance of the species-specific variants. Red bar indicates taxon temperature range in the AOM dataset considering this 15% rule.
