# Supplementary material for: Significance of gene variants for the functional biogeography of the near-surface Atlantic Ocean microbiome
Source: Nat Commun. 2022 Jan 24;13:456. doi: 10.1038/s41467-022-28128-8 (PMC8786918; doi:10.1038/s41467-022-28128-8)
Supplement: Supplementary file 3 — Description of Additional Supplementary Files [file 41467_2022_28128_MOESM3_ESM.docx]

Description of Additional Supplementary Files

Title: Supplementary Data 1

Description: 1 Log-fold change (l2) and Benjamini-Hochberg adjusted p-values of differential abundance (DESeq2) analysis of selected pathways (amino acid, oligo- and monosaccharide transporters, nitrogen metabolism, vitamin B12 and B1 synthesis, phytosynthesis and CAZymes) between functional profile clusters 1, 2 and 3 of the AOM.

Title: Supplementary Data 2

Description: 1. Taxonomy of 158 species/genomes and their temperature range, temperature of maximum abundance and lowest and highest temperature of occurrence

2. Number of variants, temperature range and standard deviation of highly abundant KOs of prominent taxa of the AOM.
