# Supplementary material for: Significance of gene variants for the functional biogeography of the near-surface Atlantic Ocean microbiome
Source: Nat Commun. 2022 Jan 24;13:456. doi: 10.1038/s41467-022-28128-8 (PMC8786918; doi:10.1038/s41467-022-28128-8)
Supplement: Supplementary file 6 — Reporting Summary [file 41467_2022_28128_MOESM6_ESM.pdf]

nature research

Corresponding author(s): Meinhard Simon

Last updated by author(s): 2021/11/30

# Reporting Summary

Nature Research wishes to improve the reproducibility of the work that we publish. This form provides structure for consistency and transparency in reporting. For further information on Nature Research policies, see our Editorial Policies and the Editorial Policy Checklist.

## Statistics

For all statistical analyses, confirm that the following items are present in the figure legend, table legend, main text, or Methods section.

| n/a | Confirmed | |
|---|---|---|
| ☐ | ☒ | The exact sample size (*n*) for each experimental group/condition, given as a discrete number and unit of measurement |
| ☐ | ☒ | A statement on whether measurements were taken from distinct samples or whether the same sample was measured repeatedly |
| ☐ | ☒ | The statistical test(s) used AND whether they are one- or two-sided<br>*Only common tests should be described solely by name; describe more complex techniques in the Methods section.* |
| ☐ | ☒ | A description of all covariates tested |
| ☐ | ☒ | A description of any assumptions or corrections, such as tests of normality and adjustment for multiple comparisons |
| ☐ | ☒ | A full description of the statistical parameters including central tendency (e.g. means) or other basic estimates (e.g. regression coefficient) AND variation (e.g. standard deviation) or associated estimates of uncertainty (e.g. confidence intervals) |
| ☐ | ☒ | For null hypothesis testing, the test statistic (e.g. *F*, *t*, *r*) with confidence intervals, effect sizes, degrees of freedom and *P* value noted<br>*Give P values as exact values whenever suitable.* |
| ☒ | ☐ | For Bayesian analysis, information on the choice of priors and Markov chain Monte Carlo settings |
| ☐ | ☒ | For hierarchical and complex designs, identification of the appropriate level for tests and full reporting of outcomes |
| ☐ | ☒ | Estimates of effect sizes (e.g. Cohen's *d*, Pearson's *r*), indicating how they were calculated |

*Our web collection on statistics for biologists contains articles on many of the points above.*

## Software and code

Policy information about availability of computer code

| | |
|---|---|
| Data collection | Illumina reads were trimmed using Trimmomatic 0.3648 (ADAPTER:2:30:10 SLIDINGWINDOW:4:25 MINLEN:100). The high quality (HQ) reads were assembled using metaSPAdes 3.11.149,50. Gene-coding sequences of the assembled contigs were predicted using Prodigal 2.6.2 in meta-mode51. To generate a gene catalogue, gene sequences were clustered using USEARCH 10.0.2452 (-cluster_fast–id 0.95).<br>Non redundant gene sequences were taxonomically classified using Kaiju 1.653 (-greedy mode with 5 allowed substitutions and e-value 10e-5) with the Refseq nr (May 2018) and ProGenomes database iGene functions were assigned to AOM sequences using the Kyoto Encyclopedia of Genes and Genomes (KEGG) online annotation tool GhostKOALA55 (https://www.kegg.jp/ghostkoala/) using the prokaryotic, eukaryotic and viral KEGG gene database (release 86) and default settings. In addition, AOM sequences were translated to amino acid sequence subsequently searched against the CAZy database (version: 2018-07-31) using DIAMOND56 0.9.30.131 blastx (--more-sensitive mode) to identify CAZymes57. To check for redundancy with genes of the Tara Ocean data set, sequences of the AOM-GC were searched against the Tara-Ocean OM-RGC.v29 using BLASTN (cutoff e-value 10e-10 and ≥95% sequence identity). |
| Data analysis | To acquire gene abundance data, HQ Illumina reads longer than 75 bp were mapped to the AOM sequences using bowtie258 2.3.5 (--very-sensitive-local mode). SAMtools59 version 1.9-58-gbd1a409 was used to convert the SAM alignment file to read abundance tables. |

For manuscripts utilizing custom algorithms or software that are central to the research but not yet described in published literature, software must be made available to editors and reviewers. We strongly encourage code deposition in a community repository (e.g. GitHub). See the Nature Research guidelines for submitting code & software for further information.

## Data

Policy information about availability of data

All manuscripts must include a data availability statement. This statement should provide the following information, where applicable:

- Accession codes, unique identifiers, or web links for publicly available datasets
- A list of figures that have associated raw data
- A description of any restrictions on data availability

Sequence data generated in this study have been deposited in the European Nucleotide Archive69 (ENA) under the INSDC accession number PRJEB34453 [https://www.ebi.ac.uk/ena/browser/view/PRJEB34453] using the data brokerage service of the German Federation for Biological Data70 (GFBio), in compliance with the Minimal Information about any (X) Sequence (MIxS) standard71. Environmental data from the cruise are available in the supplement and on PANGEA under the accession number PANGAEA.906247 [https://doi.pangaea.de/10.1594/PANGAEA.906247]. The Atlantic Ocean Reference Gene Catalogue (AOM-RGC), assembled contigs and predicted genes are available at https://service.icbm.uni-oldenburg.de/data/AOM_data/.
The assembly pipeline as well as scripts used for dataset generation and analysis are available at https://github.com/LeonDlugosch/Atlantic-Ocean-Metagenomes.

# Field-specific reporting

Please select the one below that is the best fit for your research. If you are not sure, read the appropriate sections before making your selection.

☐ Life sciences ☐ Behavioural & social sciences ☒ Ecological, evolutionary & environmental sciences

For a reference copy of the document with all sections, see nature.com/documents/nr-reporting-summary-flat.pdf

# Ecological, evolutionary & environmental sciences study design

All studies must disclose on these points even when the disclosure is negative.

| | |
|---|---|
| Study description | Metagenomic analysis of samples collected at 22 stations at 20 m depth in the Atlantic and Southern Ocean between 62°S and 47°N covering all relevant biogeographic provinces. |
| Research sample | The locations of the samples were chosen to obtain representative samples from all biogeographic provinces along the transect from Antarctic to temperate north Atlantic waters. Constraints were the prefixed itinerary of the reseach vessel. Due to limited sequencing and analytical capacities only samples from 20 m depth were included in this study. All metadata used in this study stem from the 20 m samples. For the metagenomic analyses ee targeted only the prokaryotic and picoeukaryotic microbes of the water samples and therefore obtained them by prefiltering the samples through 3.0 µm and collecting them on 0.2 µm filters.<br>Environmental variables as metadata were also analysed (e.g. salinity, water temperature, chlorophyll, particulate organic carbon, inorganic nutrients, particulate nitrogen) |
| Sampling strategy | THe strategy was to include all biogeographic provinces along this transect in the set of stations. Samples at 20 m depth were collected with 12 L Niskin bottles mounted on a rosette sampler. Sample size of 40 L was necessary to collect enough microorganisms for extracting enough DNA for subsequent sequencing and bioinformatic analysis. |
| Data collection | Data were generated from the metagenomes sequenced on an Illumina HiSeq 25000 instrument. Responsibel Persons are Rolf Daniel and Anja Poehlein. Biogeochemical and microbial data were generated from the samples collected and are available at https://doi.pangaea.de/10.1594/PANGAEA.906247. Environmental data af the stations were recorded by the Research Vessel Polarstern and are published (hdl:10013/epic.40372, hdl:10013/epic.40370) https://doi.org/10.1594/PANGAEA.802809 -, https://doi.org/10.1594/PANGAEA.802810. Responsible person is Meinhard Simon |
| Timing and spatial scale | Oceanigraphic cruises are planned far ahead and the planning sets the general time frame. This was also the case for our cruises ANT XXVIII/4 and -/5. Hence and to meet the cruise schedule, sampling at the stations in the Southern Ocean started on March 16, 2012 and ended at the northernmost station in the Atlantic on May 11, 2012. Te entire transect covered 13,000 km. |
| Data exclusions | only unreliable sequencing data not passing our quality checks were excluded from further analyses. |
| Reproducibility | The metagenomic analysis of samples from a single station are based on a single sample. Due to logistic and financial constraints replicate samples could not be taken. But this procedure is quite normal in these types of analyses. |
| Randomization | this procedure is never applied in any microbial oceanographic study. For such studies it is always important to know the origin and location of the samples and depth at which they were collected. |
| Blinding | this procedure is never applied in any microbial oceanographic study and would not yield better results than analysing identified samples |

Did the study involve field work? ☒ Yes ☐ No

## Field work, collection and transport

| | |
|---|---|
| Field conditions | Oceanographic study,between the Southern Ocean and the North Atlantic based on samples collected by a rosette sampler from RV Polarstern, rough to smooth sea conditions from sea surface temperatures around 0°C to 28°C |

| | |
|---|---|
| Location | See Figure 1 and list of 22 stations in the supplement |
| Access & import/export | All work was in international waters and none in any EEZ. So there was no need for any application for sample collection in EEZ. We only dealt with frozen and no live samples. Germany did not require any import statement for frozen samples incuding only microbes. |
| Disturbance | Collection of water in the middle of the Southern and Atlantic Ocean does not cause any disturbance (except chasing a few pelagic birds) |

# Reporting for specific materials, systems and methods

We require information from authors about some types of materials, experimental systems and methods used in many studies. Here, indicate whether each material, system or method listed is relevant to your study. If you are not sure if a list item applies to your research, read the appropriate section before selecting a response.

## Materials & experimental systems

| n/a | Involved in the study |
|---|---|
| ☒ | ☐ Antibodies |
| ☒ | ☐ Eukaryotic cell lines |
| ☒ | ☐ Palaeontology and archaeology |
| ☒ | ☐ Animals and other organisms |
| ☒ | ☐ Human research participants |
| ☒ | ☐ Clinical data |
| ☒ | ☐ Dual use research of concern |

## Methods

| n/a | Involved in the study |
|---|---|
| ☒ | ☐ ChIP-seq |
| ☒ | ☐ Flow cytometry |
| ☒ | ☐ MRI-based neuroimaging |
